# Esterification of Glycerol With Oleic Acid Over Hydrophobic Zirconia-Silica Acid Catalyst and Commercial Acid Catalyst: Optimization and Influence of Catalyst Acidity

**DOI:** 10.3389/fchem.2019.00205

**Published:** 2019-04-10

**Authors:** Pei San Kong, Yolande Pérès, Wan Mohd Ashri Wan Daud, Patrick Cognet, Mohamed Kheireddine Aroua

**Affiliations:** ^1^Sime Darby Research Sdn. Bhd., Pulau Carey, Malaysia; ^2^Laboratoire de Génie Chimique, CNRS, INP, UPS, Université de Toulouse, Toulouse, France; ^3^Department of Chemical Engineering, Faculty of Engineering, University of Malaya, Kuala Lumpur, Malaysia; ^4^Centre for Carbon Dioxide Capture and Utilization, School of Science and Technology, Sunway University, Bandar Sunway, Malaysia; ^5^Department of Engineering, Lancaster University, Lancaster, United Kingdom

**Keywords:** hydrophobic silica-zirconia based catalyst, optimisation, acidity, selectivity, esterification, glycerol

## Abstract

Catalytic esterification of glycerol with oleic acid (OA) was optimized over hydrophobic mesoporous zirconia-silica heterogeneous acid catalyst (ZrO_2_-SiO_2_-Me&Et-PhSO_3_H) and benchmarked with commercial catalysts (Aquivion and Amberlyst 15) in order to examine the effect of catalyst acidity on conversion, yield and product selectivity. The process optimisation results showed an 80% conversion with a 59.4% glycerol mono-oleate (GMO) and 34.6% glycerol dioleate (GDO) selectivities corresponding to a combined GMO and GDO selectivity of 94.8% at equimolar OA-to-glycerol ratio, 160°C reaction temperature, 5 wt% catalyst concentration with respect to the OA weight and 4 h reaction time. This work reveals that the hydrophobic and mild acidic ZrO_2_-SiO_2_-Me&Et-PhSO_3_H catalyst outperformed Amberlyst 15 and Aquivion with a yield of 82% and GMO selectivity of 60%. It is found that catalyst acidity is a key parameter for catalytic activity and conversion rate. Nevertheless, high acidity/acid strength reduced the product yield in the glycerol esterification of OA.

## Introduction

Glycerol mono, di-oleates (GMO, GDO) are lipids with amphiphilic, non-ionic and excellent emulsifying properties that widely applied in food, cosmetic, and pharmaceutical industries, and aqueous fiber finishing (Thengumpillil et al., 2002; Macierzanka and Szelag, 2004). GMO featuring a polar head group and a non-polar hydrocarbon chain which exhibits significant amphiphilic properties. Therefore, GMO could be self-assembled into different liquid crystalline structures under varying conditions of temperature and solvent composition (Kulkarni et al., 2011). The actively growing industries such as personal care, pharmaceuticals, and lubricants are the main outlets for GMO. Nevertheless, the demand of GMO is correlated to personal care or lubricant market due to the gradual slowdown in the food and plastics sectors (Frost Sullivan Research Service, 2014). Meanwhile, GDO is mainly used in drug delivery applications and as safe plasticizers for the polymer industry (Barauskas et al., 2006; Zhang et al., 2017).

The esterification reaction between glycerol and fatty acids can be an economic process. Glycerol can be obtained from low costs feedstock such as co-product in the biodiesel industry. Extensive research investigations on glycerol conversion to value-added chemicals have been shepherded due to surplus glycerol production and its inevitably low value (Quispe et al., 2013; Kong et al., 2016a). Generally, acid catalytic system is employed in esterification, etherification, polymerization, dehydration, acetylation, or glycosylation reaction (Kong et al., 2015, 2016b; Karam et al., 2017). The catalytic esterification of glycerol with large molecular size of oleic acid (OA) can produce numerous high commercial value of glycerol mono-, di-, and tri-oleate (GMO, GDO, and GTO).

Mesoporous silica, metal oxide, modified zeolites, heteropolyacids-supported catalysts (Wee et al., 2013) and ion-exchange resins (Amberlyst 15, Amberlyst 16, Amberlyst 31) (Åkerman et al., 2011), double-metal cyanide complexes (Kotwal et al., 2011), hydrotalcite (Hamerski and Corazza, 2014; Hamerski et al., 2016) and sulfated metal oxides catalysts (Kong et al., 2015) have been investigated as potential catalysts for glycerol esterification with OA. It was reported that the use of DMC complex allowed to obtain a conversion of 63.4% together with a 67.3% GMO selectivity at reaction temperature (180°C), catalyst concentration (8 wt%), and equimolar glycerol-to-OA ratio in 8 h reaction time (Kotwal et al., 2011). Notably, the outstanding catalytic performance was not only ascribed to the available catalyst sites in enhancing the conversion but also to the catalyst structure for maximize the selectivity of desired product. In terms of process parameters, the selectivity toward GMO and GDO formation is higher when using higher glycerol concentration, shorter reaction time, and lower reaction temperature (preferably 1:4 molar ratio of OA to glycerol, 3–6 h, <180°C). On the contrary, the formation of GTO can be achieved by increasing reaction time and operating temperature at higher OA environment (3:1 molar ratio of OA to glycerol, >10 h, >180°C) (Kong et al., 2015).

It has been reported that catalyst surface structure together with operating parameters play an important role in controlling the selectivity of products, whereby the yields rely mainly upon catalyst properties, amongst which hydrophobicity is of great importance. Consequently, this work examines the optimization study of hydrophobic-enhanced ZrO_2_-SiO_2_-Me&Et-PhSO_3_H catalyst under various operating conditions such as reactants molar ratio, catalyst concentration, reaction temperature, and reaction time. The novelty of this work is to examine the influence of ZrO_2_-SiO_2_-Me&Et-PhSO_3_H catalyst acidity in yield and product selectivity in comparison to the commercial available catalysts with higher acidity (Amberlyst 15; Aquivion) at optimized conditions as effect of catalyst acidity has not been insight studied in literatures. Furthermore, this work investigates the combination catalyst properties as such hydrophobicity and acidity strength in order to obtain desired product yield and color. Nevertheless, this work also provides interaction effect of important operating parameters to acquire higher product selectivity.

## Experimental

### Materials

Zirconium hydroxide powder [Zr(OH)_4_, 97% purity, Sigma-Aldrich], ethanol (99%), ammonia solution (NH_4_OH, 25%, Sigma-Aldrich), tetraethyl orthosilicate (TEOS, 98%, Sigma-Aldrich), trimethoxymethylsilane (TMMS, 98%, Sigma-Aldrich), dry toluene (99%, Sigma-Aldrich), 2-(4-Chlorosulfonylphenyl) ethyltrimethoxysilane (CSPETS, 50% in dichloromethane, Fisher Scientific) and sulfuric acid solution (H_2_SO_4_, 99.99%) were used to synthesize ZrO_2_-SiO_2_-Me&Et-PhSO_3_H hydrophobic-enhanced catalyst. Zirconium (IV) propoxide, Zr(OCH_2_CH_2_CH_3_)_4_ precursor (70% in 1-propanol, Sigma-Aldrich), 1-propanol (99.7%, Sigma-Aldrich), and 0.5 M aqueous H_2_SO_4_ were utilized to produce sol-gel method-prepared catalyst (${\text{SO}}_{4}^{2-}$/ZrO_2__solgel_). The precipitation method prepared-catalyst, (${\text{SO}}_{4}^{2-}$/ZrO_2__precipitation_) was synthesized using zirconium oxychloride precursor, (ZrOCl_2_.8H_2_O, 99.5%, Sigma-Aldrich) and sodium hydroxide solution (NaOH, Sigma-Aldrich). ${\text{SO}}_{4}^{2-}$/ZrO_2__commercial_ catalyst was prepared by using commercial available Zr(OH)_4_ and aqueous H_2_SO_4_. Commercial catalyst such as Amberlyst 15 (Sigma-Aldrich) and Aquivion (Solvay) were used for comparison study. Reactants glycerol (≥99.5%) and OA (technical grade, 90%) were purchased from Sigma-Aldrich. All the analytical standard reagents such as monoolein (≥99%), diolein (≥99%), and triolein (≥99%) were purchased from Sigma-Aldrich for quantitative product formation analysis. Analytical grade solvents such as acetonitrile (ACN), methanol (MeOH) and tetrahydrofuran (THF) were used as mobile phase and trifluoroacetic acid (TFA) was used as mobile phase additive.

### Catalyst Preparation

The preparation steps of catalyst featured with hydrophobic surface, which synthesized with tailored amount of TMMS hydrophobic agent (ZrO_2_-SiO_2_-Me&Et-PhSO_3_H) were described in our published work (Kong et al., 2018). ZrO_2_ was added into ethanol under vigorous mixing condition at ambient temperature for 30 min. Twelve milliliters of NH_4_OH and 4 ml of TEOS were successively added and stirred for 24 h to generate white silica suspension environment. The produced ZrO_2_-SiO_2_ was then filtered, rinsed with ethanol and dried overnight under vacuum at room temperature. Meanwhile, modification of ZrO_2_-SiO_2_ to higher hydrophobicity level together with functionalization of sulfonic acid group into ZrO_2_-SiO_2_ surface were carried out using TMMS and CSPETS (hydrophobic and surface initiating agents) (Mobaraki et al., 2014). The functionalized catalyst (ZrO_2_-SiO_2_-Me&Et-PhSO_2_Cl) was then washed with toluene and distilled water. Eventually, the functionalized ZrO_2_-SiO_2_-Me&Et-PhSO_2_Cl solids were suspended in H_2_SO_4_ solution for 2 h (0.5 M, 5 ml) for cation-anion exchange of Cl^−^ with H^+^ cation to form of ZrO_2_-SiO_2_-Me&Et-PhSO_3_H. It was washed several times with water and dried overnight under vacuum at room temperature. The formed ZrO_2_-SiO_2_-Me&Et-PhSO_3_H catalyst for glycerol catalytic esterification reaction.

### Catalyst Characterization

N_2_ physisorption method [BELSORP-max analyser (Japan)] was used to analyse the textural properties of catalysts by degassing the catalyst samples under vacuum condition at 200°C for 5 h. Meanwhile, the particle size distributions of catalysts were measured by dry Malvern MS3000 particle sizer at pressure of 2 bar. The acidity of catalyst (mmol/g) was determined by acid-base titration. Forty–Fifty milligrams of catalyst sample was degassed at 120°C for 3 h and subsequently suspended in 25 ml of NaCl (2 M) and stirred for 24 h at room temperature to achieve equilibrium and then to be titrated with 8.38 × 10^−3^ M NaOH solution. The fresh and spent catalyst was evaluated by hydrophobicity levels (water contact angle method) using KRUSS DSA100, N_2_ physisorption, and Field Emission Scanning Electron Microscope (FESEM) at 1–30 kV acceleration voltage by instrument model JSM-7100F.

### Catalytic Reaction

The catalytic esterification reaction of glycerol with OA was performed in a 250 ml batch reactor equipped with a temperature indicator and connected to a condenser and a vacuum system at 160°C, equimolar ratio of reactant and constant acidity (1.55 mmol H^+^) for 8 h. The samples were analyzed using high performance liquid chromatography coupled to refractive index detection (HPLC-RI) through an isocratic method, equipped with Gemini C18 11OA column (100 × 2 mm × 3 μm). The OA and GMO groups of the sample were separated using a mobile phase consisted of ACN/water (80:20 v/v) with 0.1% TFA (v/v of total mobile phase). Meanwhile, GDO and GTO groups were separated using ACN/MeOH/THF (40:40:20 v/v/v) (Lee et al., 2013). The injection volume was 10 μL and the diluted samples were eluted at a 220 μL/min flow rate. The column and RI detector temperatures were set at 40°C. The conversion, yield and selectivity of the products are defined by Equations 1 to 3.

(1) $$\begin{array}{l} \text{Conversion}=\;\frac{mol_{OA_{consumed}}}{mol_{OA_{initial}}}\;\times \;100\;\% \end{array}$$

(2) $$\begin{array}{l} {\text{Yield}}_{total\;GMO,GDO,GTO}=\;\frac{mol_{total\;esters\;}}{mol_{OA_{initial}}}\;\times \;100\;\% \end{array}$$

(3) $$\begin{array}{l} {\text{Selectivity}}_{GMO}\;=\;\frac{mol_{GMO}}{mol_{total\;GMO+GDO+GTO\;}}\times \;100\;\% \end{array}$$

## Results and Discussion

### Process Optimization

**Scheme 1:**
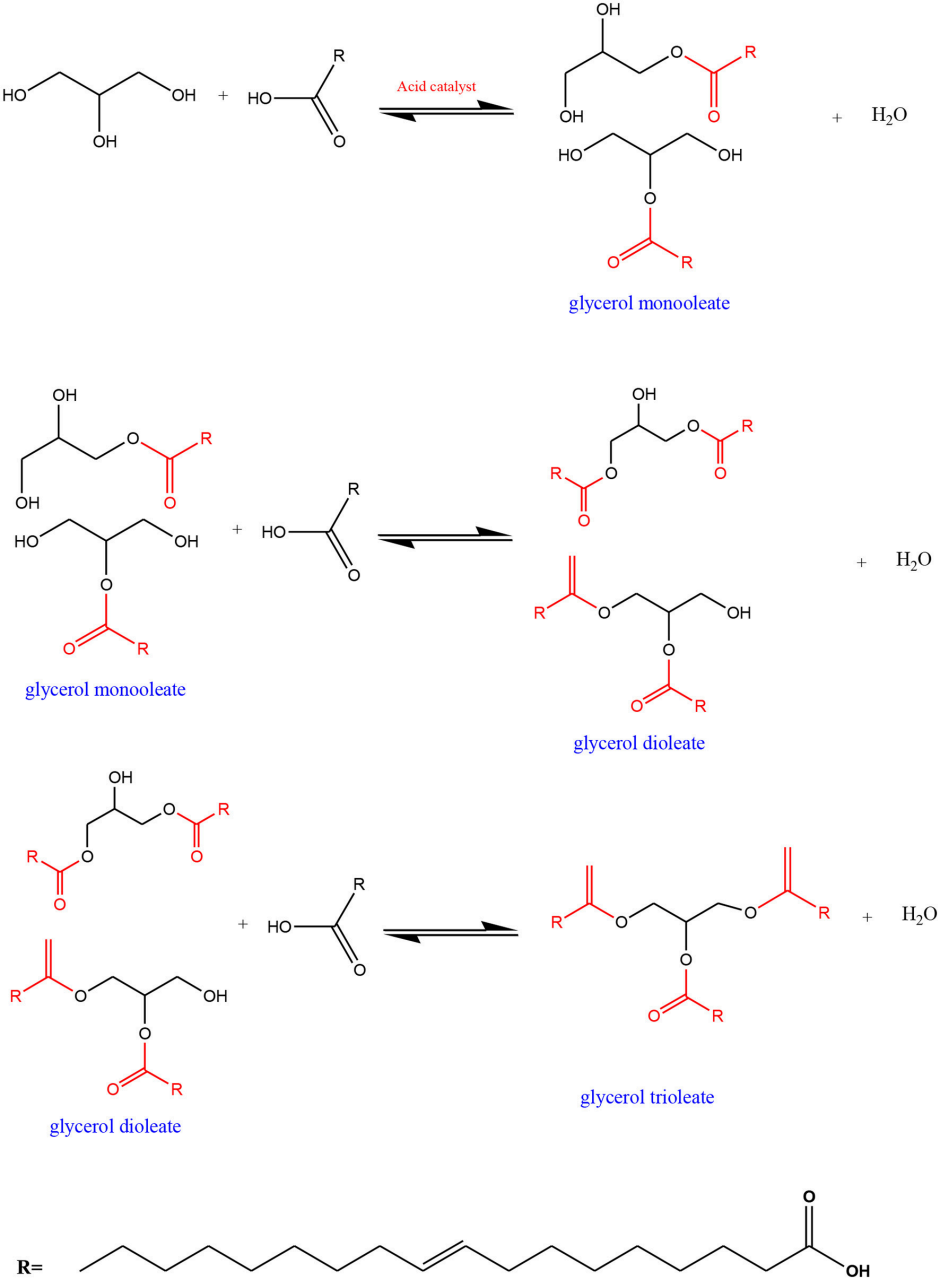
Mechanism for esterification of glycerol with OA in GMO, GDO, and GTO production.

The ZrO_2_-SiO_2_-Me&EtPhSO_3_H catalyst was used to study the effect of process operating parameters. The influences of reaction temperature, catalyst concentration, glycerol-to-OA molar ratio, and reaction time on the catalytic glycerol esterification with OA were investigated. The mass transfer limitation was evaluated prior to investigating the process variables to ensure that the esterification process was reaction controlled. Mechanism for esterification of glycerol with OA in GMO, GDO, and GTO production is presented in Scheme 1.

#### Effects of Mass Transfer

Mass transfer limitation was assessed at the reaction temperature of 100°C prior to temperature optimisation. The experiments were carried out at an equimolar OA-to-glycerol ratio, 100°C reaction temperature, 480 min and 3 wt% catalyst concentration with respect to the OA weight and solvent-less reaction conditions during mass transfer limitation study. The reaction yield and selectivity catalyzed by ZrO_2_-SiO_2_-Me&EtPhSO_3_H catalyst were evaluated in two different stirring speeds (300 and 650 rpm). The high stirring speed 650 rpm resulted in a slightly increased yield (from 35.3 to 37.4%) compared with the 300 rpm reaction speed. The selectivity of GDO and GTO also slightly increased compared with the reaction at 300 rpm, but the difference was insignificant. Therefore, the maximum stirring speed of 650 rpm was proposed for further testing of process variables in the presence of ZrO_2_-SiO_2_-Me&EtPhSO_3_H catalyst to eliminate the external mass transfer resistance. It was reported that external mass transfer resistance can be completely eliminated when a high stirring speed is applied, and external diffusion negligibly affects the overall reaction rate (Nanda et al., 2014).

#### Effects of Reaction Temperature

**Figure 1:**
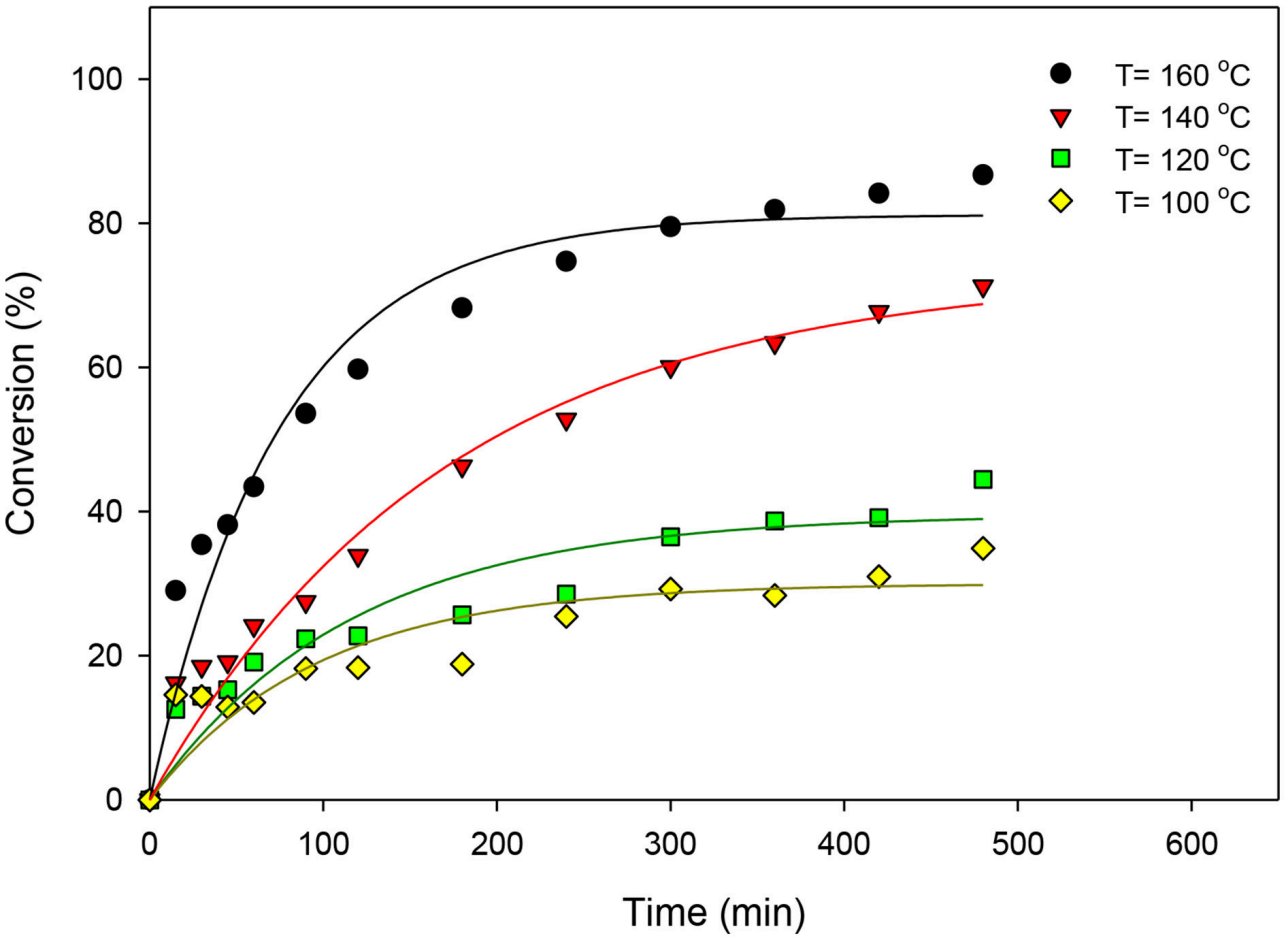
Effects of reaction temperature on the catalytic esterification of glycerol with OA using ZrO_2_-SiO_2_-Me&EtPhSO_3_H catalyst.

ZrO_2_-SiO_2_-Me&EtPhSO_3_H catalyst was used to study the effects of reaction temperature. Various temperatures (100, 120, 140, and 160°C) were utilized under the stirring speed of 650 rpm, equimolar OA-to-glycerol ratio, 3 wt% catalyst concentration with respect to the OA weight and solvent-less reaction conditions. Figure 1 presents the effects of reaction temperature on the catalytic esterification of glycerol with OA. Results indicated that the conversion increased with increased reaction temperature because high temperature favors a high equilibrium product yield in a typical endothermic reaction (Trinh et al., 2018). The initial rate of esterification also increased with increased reaction temperature. The reaction temperature of 160°C yielded the highest conversion level (86.7%) in this study. On the contrary, relatively low activities were observed at the beginning of reaction at 100° and 120°C. The activation energy required for successful conversion is difficult to exceed at low temperatures because the energy possessed by the reactant molecules is low; consequently, the effective collision is decreased because the kinetic energy in the reactant molecules and potential energy of molecules are decreased (Hoo and Abdullah, 2014).

**Figure 2:**
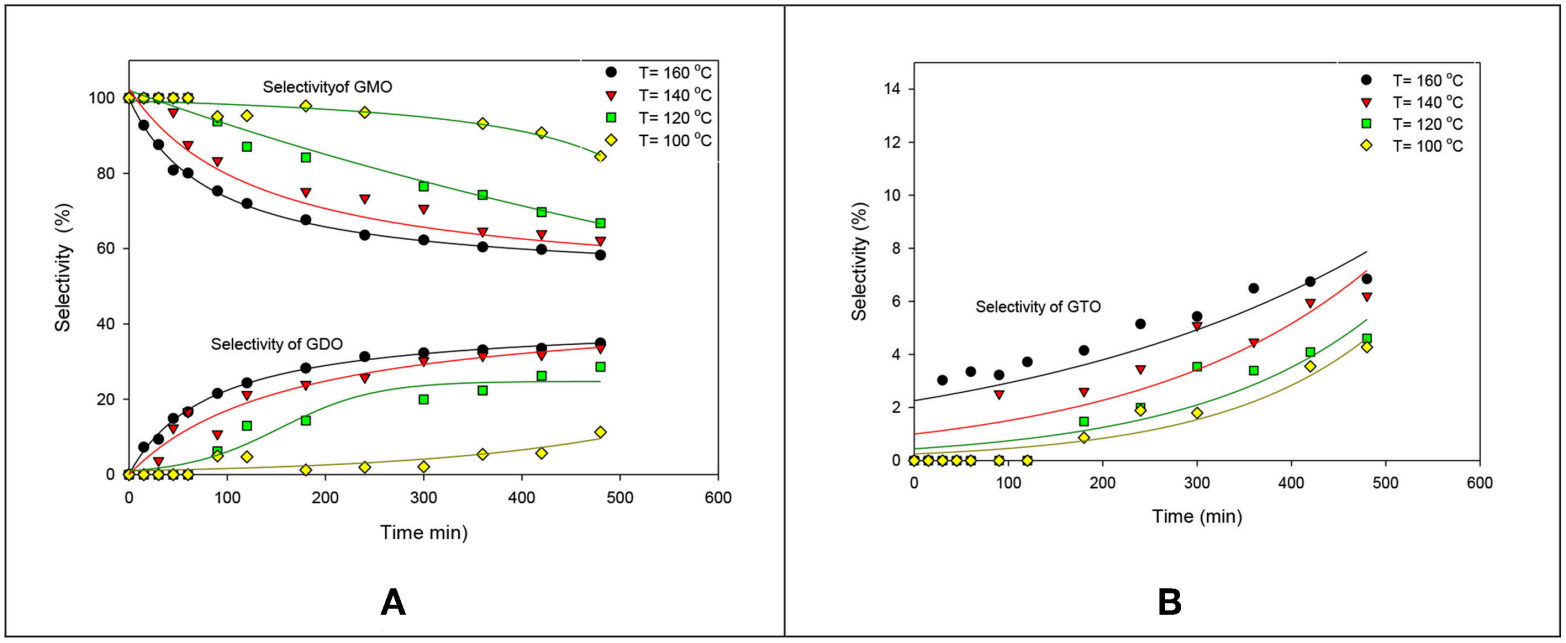
Effects of reaction temperature on the selectivities of GMO, GDO **(A)**, and GTO **(B)**.

The effects of reaction temperature on the selectivity of GMO, GDO, and GTO are shown in Figure 2. The GMO selectivity decreased by increasing the reaction temperature but both GDO and GTO selectivities increased. Notably, the selectivity percentage at 140° and 160°C were much alike, particularly at more than 360 min because ~60% of GMO and 36% of GDO were obtained. Therefore, 160°C was suggested as the optimal reaction temperature by considering the obtained conversion and selectivity under the ZrO_2_-SiO_2_-Me&EtPhSO_3_H-catalyzed esterification reaction of glycerol with OA.

The interaction effects of reaction time and reaction temperature on the conversion and selectivity of GMO. The highest conversion was obtained at 160°C after 480 min reaction time. The selectivity of GMO decreased with time and temperature. The intersection point corresponding to the highest conversion (74%) and GMO selectivity (63.6%) is obtained at 160°C and after 240 min reaction time, at equimolar OA-and-glycerol ratio and 3 wt% catalyst concentration. It is worthy to note that under these conditions the GMO and GDO combined selectivity is 91.6%.

#### Effects of the Oleic Acid-to-Glycerol Molar Ratio

**Figure 3:**
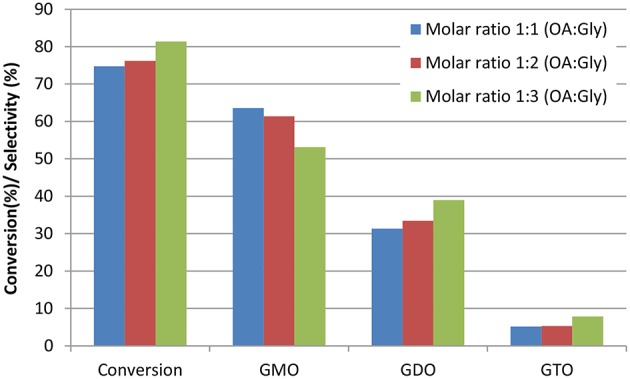
Effects of the OA-to-glycerol molar ratio on conversion and selectivity at 240 min reaction time. Conditions: catalyst concentration of OA, 3 wt%; reaction temperature, 160°C and stirring speed, 650 rpm.

The effects of excess glycerol on glycerol esterification with OA catalyzed by ZrO_2_-SiO_2_-Me&EtPhSO_3_H were investigated at the constant reaction temperature of 160°C, catalyst concentration of 3 wt%, stirring speed of 650 rpm and solvent-less reaction conditions. At 480 min reaction time, the conversion increased slightly with increased glycerol amount in the following descending order: 91.6, 89.0, and 87.5% for 1:3, 1:2, and 1:1 OA-to-glycerol molar ratios, respectively. According to Le Chatelier's principle, the glycerol esterification with OA will shift to improve products formation with increased reactant concentration. The conversion and selectivity of various GMO, GDO, and GTO at 240 min reaction time are presented in Figure 3 to evaluate the effects of excess glycerol on conversion and selectivity. The conversion of 1:1 OA to glycerol (75%) was nearly close to the 1:2 molar ratio of OA to glycerol (76%). Results revealed that the 1:3 OA-to-glycerol molar ratio produced the highest conversion of about 82% at 240 min reaction time. Similarly, Singh et al. (2013) stated that the significant reaction rate increases when the molar ratio increases from 1:2 (OA:glycerol) and insignificantly changes when excess glycerol is added at 1:6 OA-to-glycerol molar ratio.

The selectivity of GDO and GTO increased with the increased glycerol feeding ratio. Thus, the GMO selectivity was minimized by increasing the loading amount of glycerol in the catalytic esterification of glycerol with OA. It has been reported that unreacted glycerol removal is necessary despite of an equimolar OA-to-glycerol ratio of reactant was used in reaction (Konwar et al., 2016). Therefore, it can be concluded that equimolar OA-to-glycerol ratio can produce high GMO and GDO yield and equimolar ratio is suggested to obtain maximum GMO and GDO yield.

**Figure 4:**
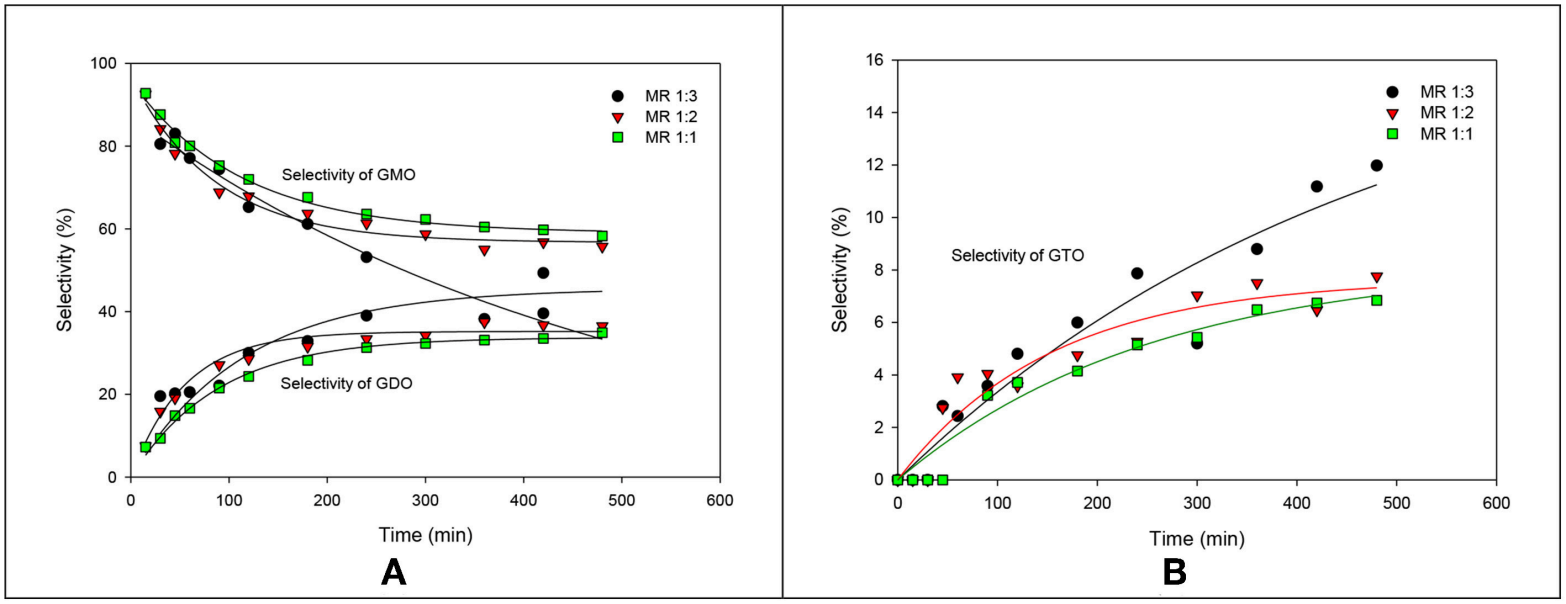
Effects of OA-to-glycerol molar ratios on the selectivities of GMO, GDO **(A)**, and GTO **(B)**.

The GMO, GDO and GTO selectivities at different molar ratios are illustrated in Figure 4. The GMO selectivity decreased with increased glycerol ratio; by contrast, increased glycerol concentrations improved GDO and GTO selectivity. Significant GDO and GTO increments were also observed at more than 240 min reaction time. This work revealed that the selectivity profiles for the OA-to-glycerol molar ratios of 1:1 and 1:2 were similar, which demonstrated that no significant effect was observed for excess glycerol amount in the glycerol:OA molar ratio range of 1–2.

**Figure 5:**
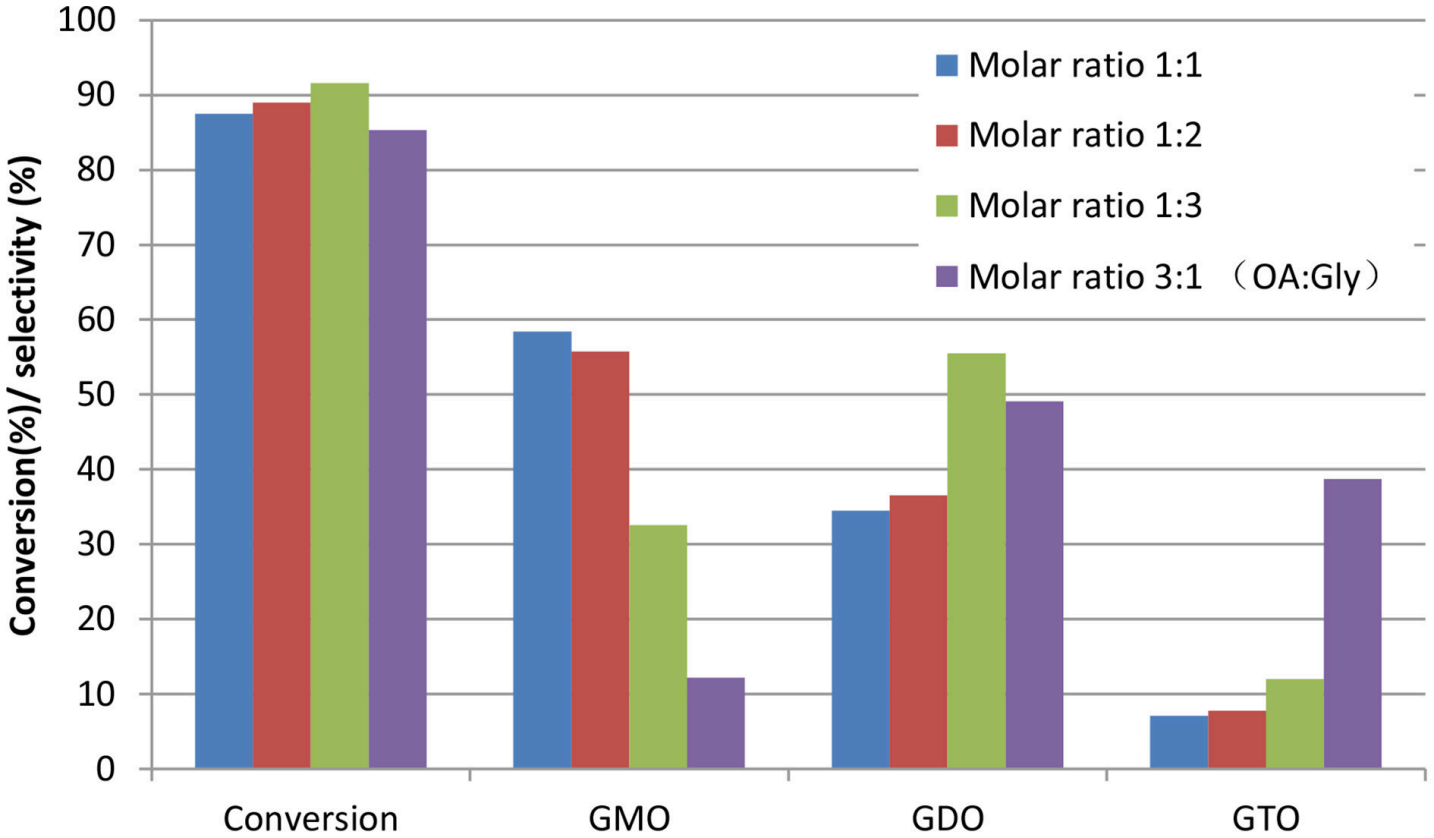
Effects of OA-to-glycerol molar ratios at 480 min reaction time. Conditions: catalyst concentration of OA, 3 wt%; reaction temperature, 160°C and speed, 650 rpm.

Glycerol esterification with OA was conducted with excess OA to compare the different reaction behavior in the OA-to-glycerol molar ratio of 3:1 at 160°C and 3 wt% catalyst concentration of OA for 480 min. Figure 5 shows the conversion and selectivity obtained at the OA-to-glycerol molar ratio with excess glycerol (1:1, 1:2, and 1:3) and OA (3:1) conditions. This work showed that excess OA caused the high formation of GTO (selectivity = 40%) and GDO (selectivity = 50%) and relatively low GMO yield. This work also confirmed that an equimolar OA-to-glycerol ratio resulted in an optimum yield of GMO, with 93% combined selectivity of GMO and GDO. The interaction effects of glycerol-to-OA molar ratio and reaction time on the conversion and selectivity of GMO are performed. It is clearly indicated that reaction time exerts stronger influence on selectivity and conversion than the molar ratio.

#### Effects of Catalyst Concentration

**Figure 6:**
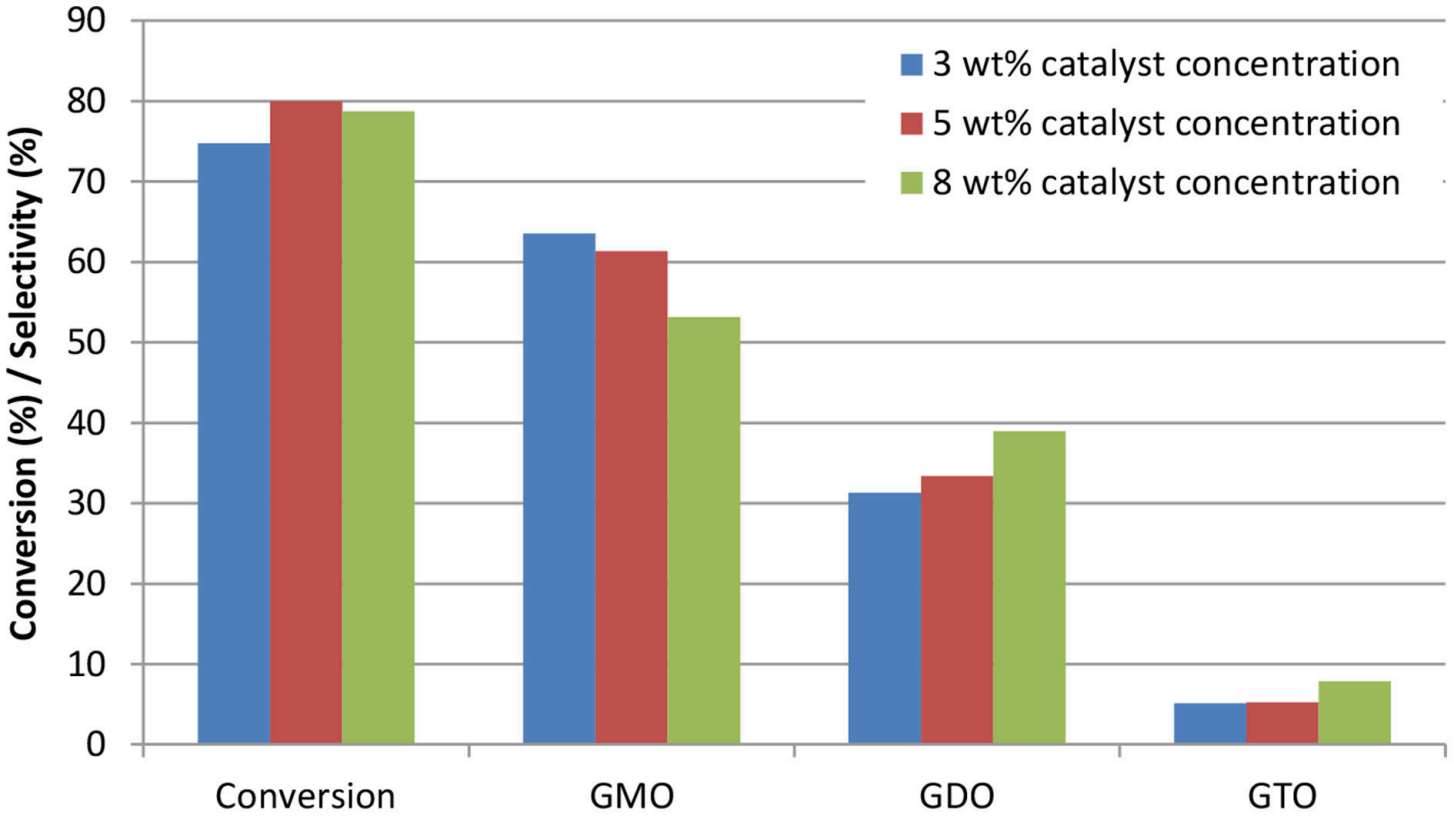
Effects of catalyst concentration on the conversion and selectivity at 240 min reaction time. Conditions: equimolar glycerol-to-OA ratio; reaction temperature, 160°C and speed, 650 rpm.

ZrO_2_-SiO_2_-Me&EtPhSO_3_H catalyst (3, 5, and 8 wt%) was used to investigate the effects of catalyst concentration on the conversion and selectivity of the catalytic glycerol esterification with OA at the constant operation parameters of 160°C, equimolar ratio and 650 rpm. Catalyst loading was calculated with respect to the weight of the limiting reactant OA. Figure 6 shows the effects of catalyst concentration on the conversion and selectivity at 240 min by using different concentrations of ZrO_2_-SiO_2_-Me&EtPhSO_3_H catalyst. Catalyst concentrations 3, 8, and 5 wt% achieved the slowest reaction rate in sequence. At 240 min reaction time, the obtained conversion was 74.7, 80.0, and 78.8% for the catalyst concentrations of 3, 5, and 8 wt%, respectively. These results proved that 5 wt% catalyst concentration was the optimal level for this catalytic study. Increasing the catalyst concentration at more than 5 wt% was not recommended because such increase does not improve the conversion. A similar trend was also reported in a previous work on glycerol esterification with palmitic acid; the conversion is unaffected beyond a certain amount of catalyst loading (Yusoff and Abdullah, 2016).

Notably, the GMO selectivity trend decreased with increased catalyst concentration; the 3 wt%-produced selectivity was 64%, which was higher than that of the 5 wt% (S_GMO_ = 61%) and 8 wt% (S_GMO_ = 53%). These findings clearly revealed that GMO was successfully converted to GDO and GTO. The increased effective interaction between the reactant molecules and GTO formation was highly attributed to the increased number of available acidic sites and acidity of the catalyst. At the end of reaction, generally after 420 min, a change in catalyst loading resulted in non-accelerated reaction rate. The conversion was insignificantly influenced with further increase in catalyst loading from 5 to 8 wt% due to the equilibrium limit (Tao et al., 2015).

**Figure 7:**
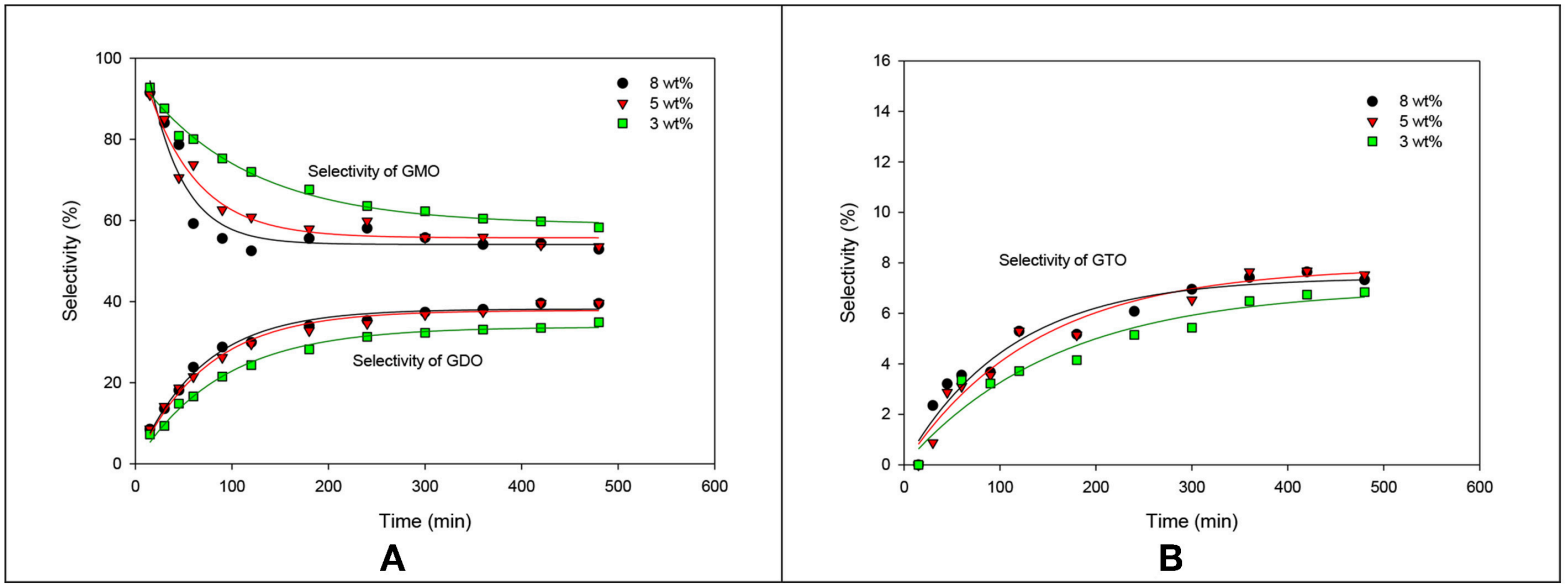
Effects of the catalyst concentration of ZrO_2_-SiO_2_-Me&EtPhSO_3_H on the selectivities of GMO, GDO **(A)**, and GTO **(B)**.

The influence of catalyst concentration on the formation trend of GMO, GDO, and GTO in terms of selectivity is elaborated in Figure 7. The selectivity profile of GMO decreased with increased catalyst concentration. The GMO selectivity curve for the 8 wt% catalyst concentration markedly decreased, particularly from 15 min to 180 min. Moreover, 5 and 8 wt% catalyst concentrations achieved high tendency to form GDO and GTO. However, the formation ratios in terms of selectivity were almost identical. In brief, the conversion acquired from ZrO_2_-SiO_2_-Me&EtPhSO_3_H-catalyzed glycerol esterification with OA was 88.2% with 53.5% of GMO and 39.6% of GDO selectivity at 5 wt% catalyst concentration, 160°C, equimolar reactant ratio and 480 min reaction time.

##### Interaction effects of catalyst concentration and reaction time

The interaction effects of catalyst concentration and reaction time on conversion and selectivity were also investigated. The aforementioned section reported that 3 wt% catalyst concentration, 240 min reaction time and an equimolar ratio of OA and glycerol resulted in 74% conversion and 63.6% of GMO selectivity (about 95% of combined selectivity of GMO and GDO).

**Figure 8:**
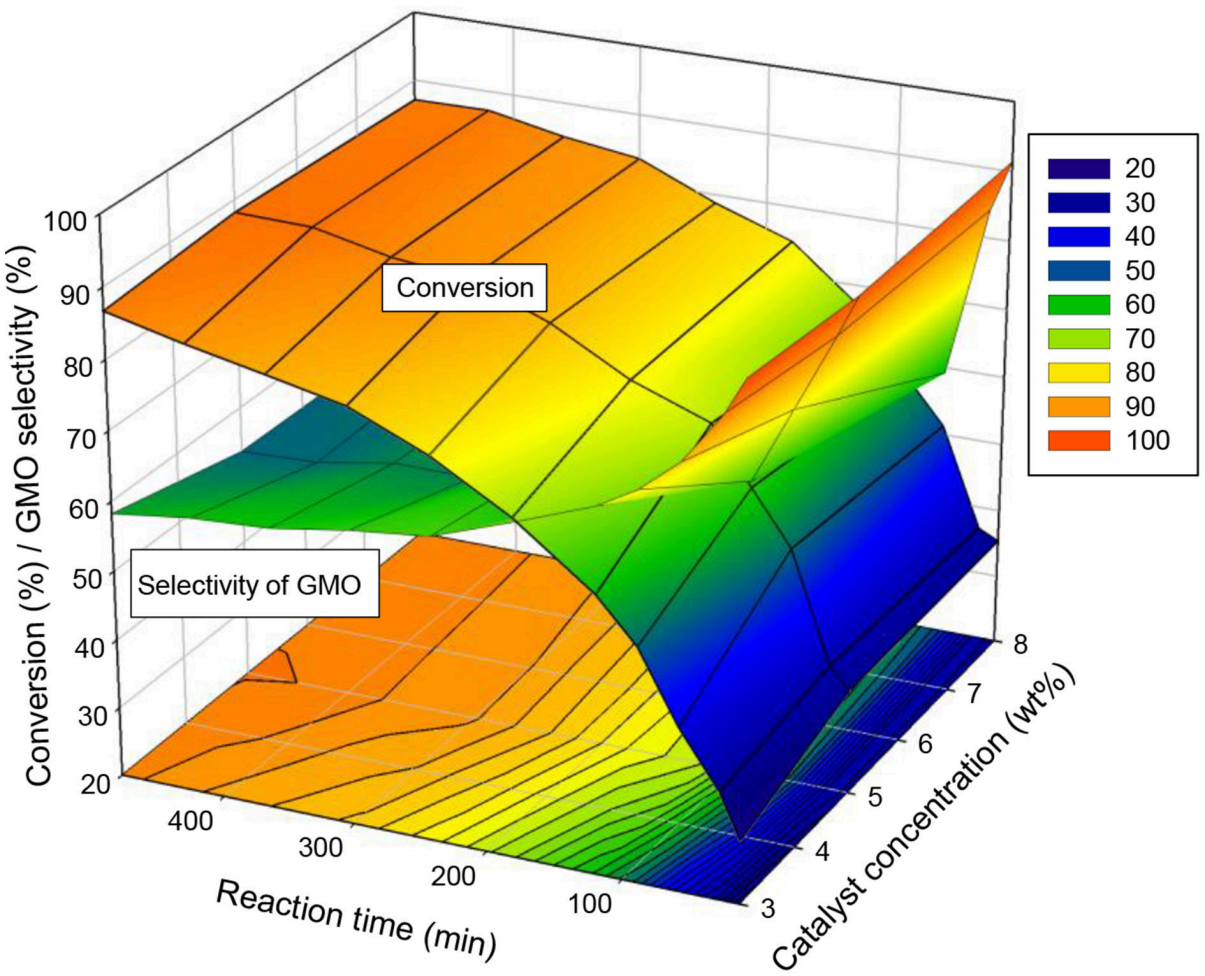
Interaction effects of catalyst concentration and reaction time on the conversion and GMO selectivity at an equimolar ratio of OA and glycerol, reaction temperature of 160°C and speed of 650 rpm.

The interaction plot in Figure 8 indicates that a short reaction time (180 min), 5 wt% catalyst concentration and an equimolar ratio of reactants allowed to achieve a conversion of 74 and 62.5% selectivity of GMO (~95.8% combined selectivity of GMO and GDO). Additionally, extending the reaction time to 240 min under the same reaction parameters (5 wt% catalyst concentration, equimolar ratio of OA to glycerol and 650 rpm) led to a conversion of 80% and about 60% selectivity of GMO, with a low combined GMO and GDO selectivity (94.8%). Consequently, 240 min reaction time was suggested for the catalytic esterification of glycerol with OA in the presence of 5 wt% of ZrO_2_-SiO_2_-Me&EtPhSO_3_H catalyst with the use of equimolar reactants.

##### Interaction effects of catalyst concentration and reaction temperature

**Figure 9:**
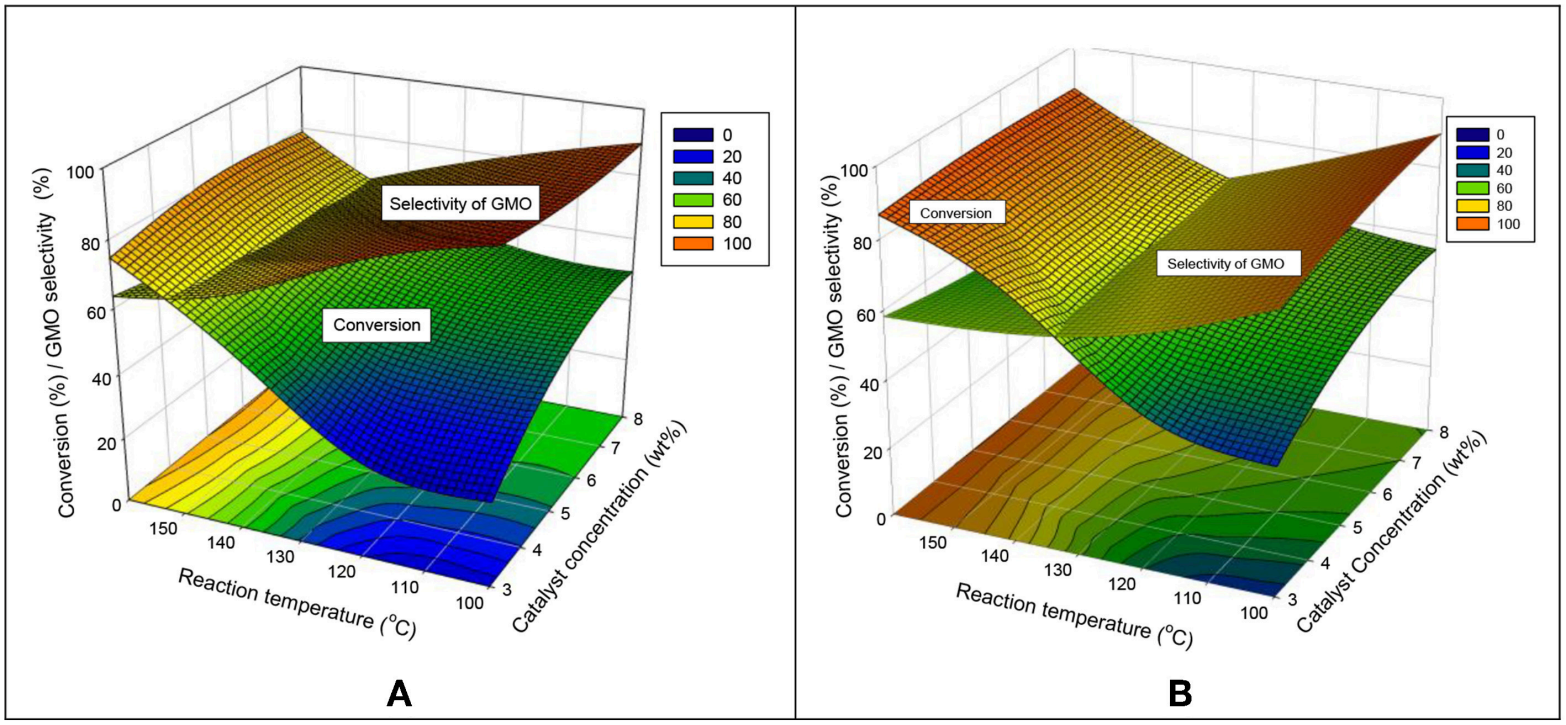
Interaction effects of catalyst concentration and reaction temperature on the conversion and GMO selectivity at **(A)** 240 and **(B)** 480 min reaction time, equimolar ratio of OA and glycerol, reaction temperature of 160°C and speed of 650 rpm.

The interaction effects of catalyst concentration and reaction temperature were studied comprehensively at 240 and 480 min in Figures 9A,B, respectively. These two response surface diagrams display a similar relation curve but different intersection points shown between the conversion and selectivity of GMO. At 240 and 480 min, the conversion can be increased in two ways: increasing the reaction temperature and the catalyst concentration. The effect of catalyst concentration was much significant at a short reaction time of 240 min.

An increased GMO selectivity can be obtained at a low reaction temperature and a high catalyst concentration. At 240 min, the GMO selectivity was highly dependent on the reaction temperature (an inclined curve was obtained). By contrast, the GMO selectivity was less dependent on the reaction temperature at a long reaction time. Figure 9B shows that at a low range of reaction temperature (100°-125°C). The GMO selectivity was high when a high loading catalyst amount was used. In conclusion, a high conversion (more than 80%) and selectivity of GMO (about 60%) can be achieved at 480 min reaction time, equimolar reactant ratio, 160°C and 650 rpm.

### Catalyst Characterizations

**Figure 10:**
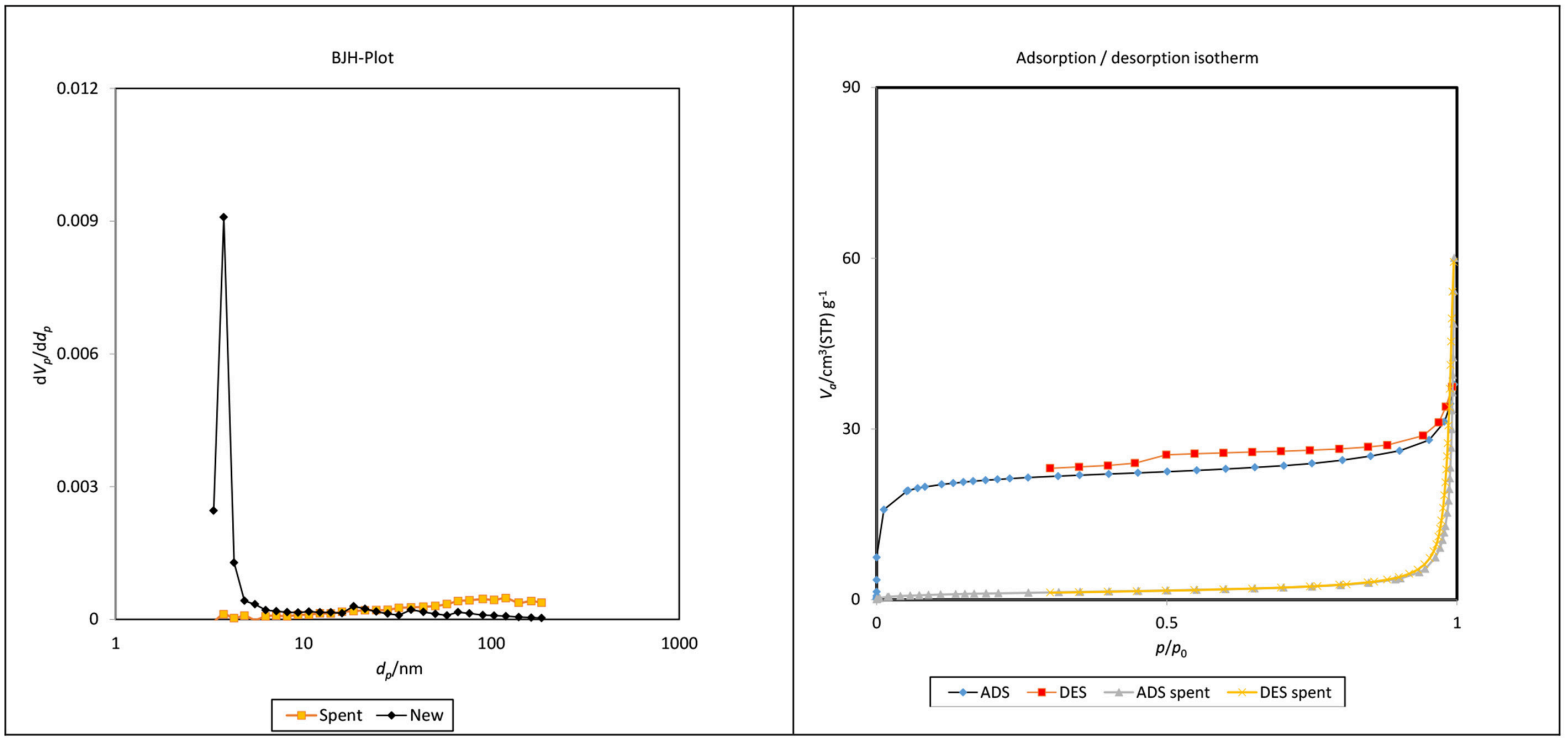
BJH plot and N_2_ adsorption–desorption isotherms of new and spent ZrO_2_-SiO_2_-Me&EtPhSO_3_H catalyst.

**Figure 11:**
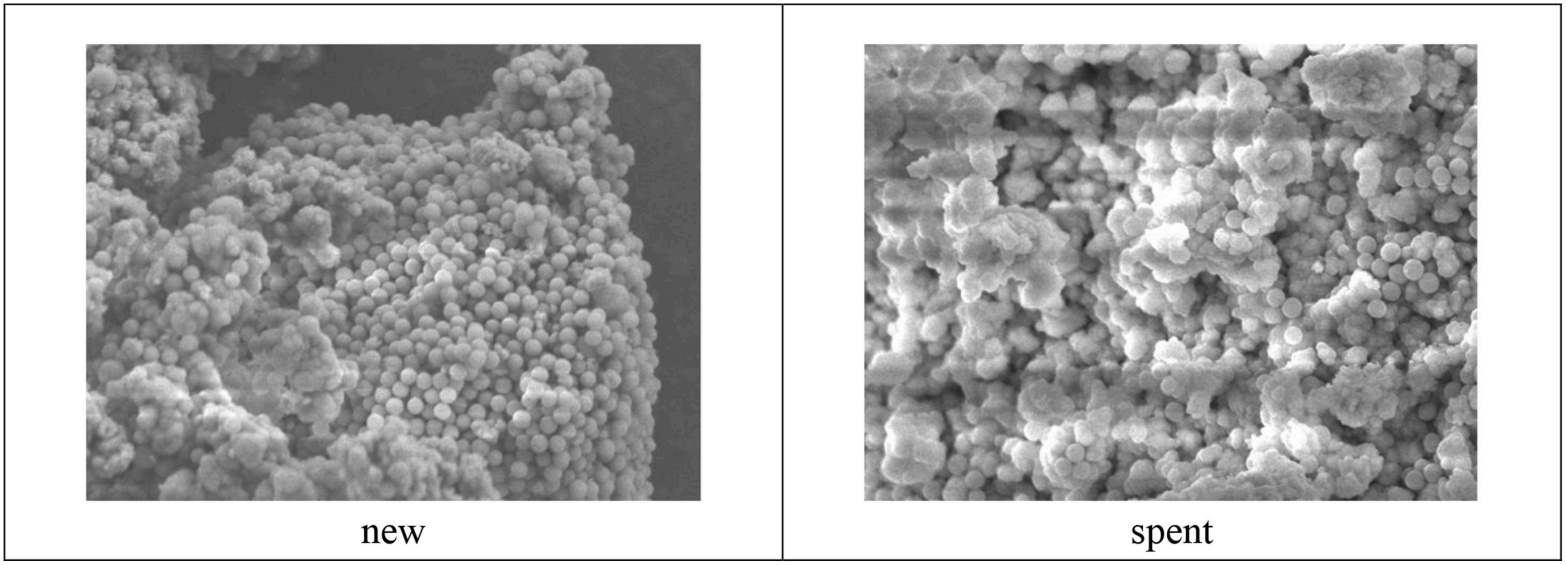
FESEM images of new and spent ZrO_2_-SiO_2_-Me&EtPhSO_3_H catalyst.

The Barrett-Joyner-Halenda (BJH) plots and N_2_ adsorption–desorption isotherms for the fresh and spent catalysts of ZrO_2_-SiO_2_-Me&EtPhSO_3_H are shown in Figure 10. The pore size distribution was unevenly distributed at the low surface area of the spent catalyst, which was most probably due to the existence of less-ordered structures of silica (Estevez et al., 2016) and the adherence of triglycerides/compounds within the pore of the spent samples (refer to FESEM image of spent catalyst, Figure 11). The contact angle analysis result of the spent catalyst was inferior (31.9°) to that of the newly developed catalyst with 41.5°.

#### Commercial Amberlyst 15 and Aquivion Characterisations and Performance Evaluations

A comparative study between ZrO_2_-SiO_2_-Me&Et-PhSO_3_H and the commercially available Amberlyst 15 and polymeric perfluorosulfonic acid (PFSA) Aquivion was performed under optimized operating reaction conditions in the glycerol esterification with OA. Aquivion PFSA is a copolymer based on tetrafluoroethylene and the sulfonyl fluoride vinyl ether catalyst from Solvay Specialty Polymers. Aquivion is a perfluorosulfonic superacid resin with a relatively high acid strength, high thermal stability and ~−12 Hammett acidity (comparable to the acid strength of H_2_SO_4_) (Fang et al., 2016). Amberlyst 15 is a conventional macroporous sulphonic ion exchange resin with 120°C thermal stability (Kong et al., 2015). The catalyst characteristics are summarized in Table S1. Although the ion exchange capacity of Aquivion PFSA (1.0 mequiv/g) used in this study is lower than that of Amberlyst 15 (4.7 mequiv/g), the Hammett acidity function of Amberlyst 15 (H_0_ = −2) is considerably lower than that of the superacid Aquivion (H_0_ = −12) (Karam et al., 2016). Thus, the influences of the different acidity strengths of catalysts were observed in the present work.

The surface area, pore volume, particle size distribution, and acidity strength of these three catalysts differed. The distribution phenomenon of each catalyst was also examined in a polar and non-polar solvent (within a layer of immiscible toluene–water phase), and result is demonstrated in Figure S1. Aquivion were located on the interface between toluene and water. Nevertheless, Aquivion exhibited an amphiphilic property because it was only located on the interface between toluene and water, unlike the Me&Et-PhSO_3_H-SiO_2_-ZrO_2_, which was distributed in the toluene phase. By contrast, Amberlyst 15 was immersed in the bottom-water phase.

**Figure 12:**
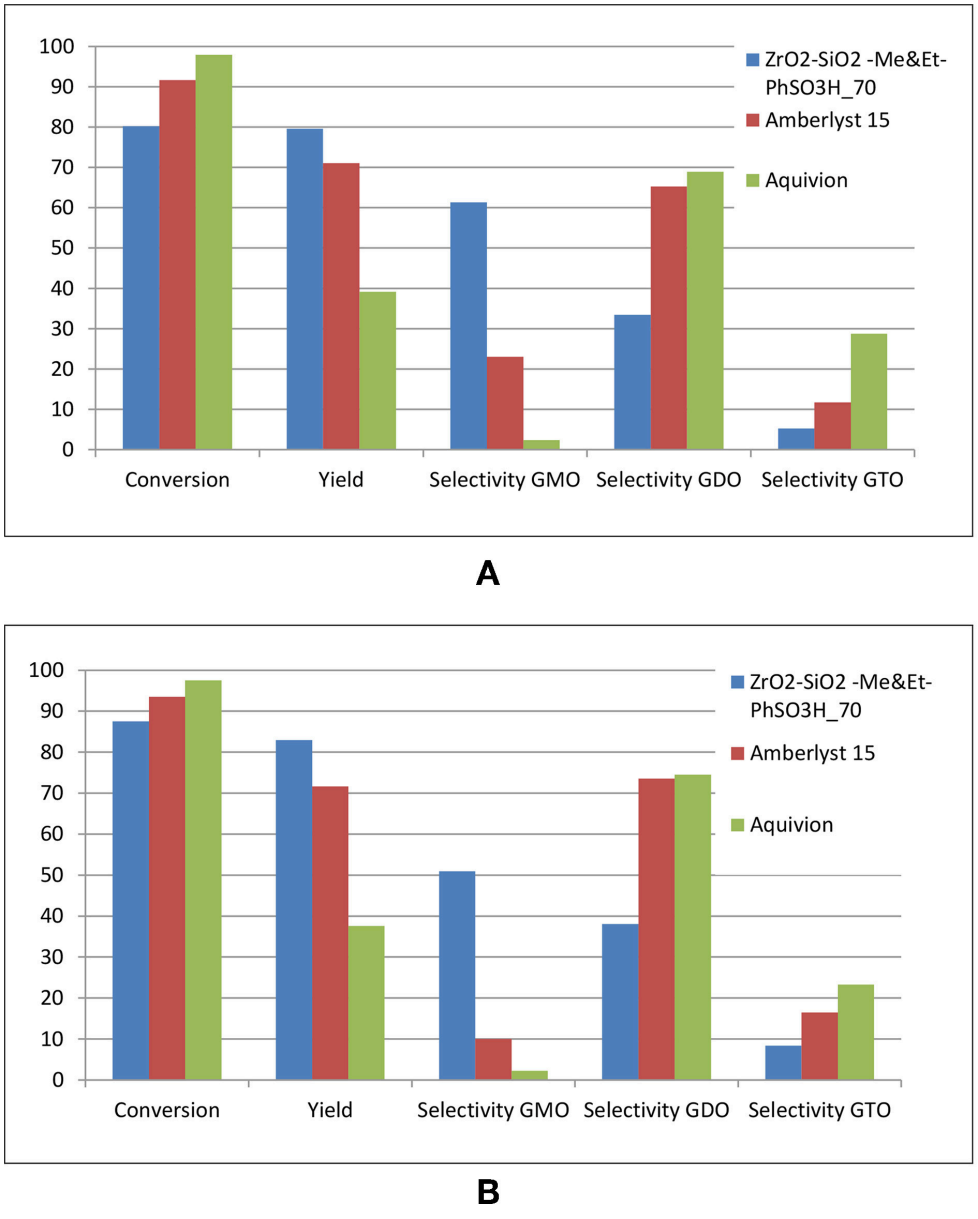
Comparison of the catalytic activities of ZrO_2_-SiO_2_-Me&EtPhSO_3_H, Amberlyst 15 and Aquivion catalysts. All reactions were conducted at the constant acidity of 1.55 mmol H^+^, equimolar ratio of OA and glycerol, reaction temperature of 160°C and stirring speed of 650 rpm for 240 min **(A)** and 480 min **(B)**.

Three sets of experiments were performed under optimized conditions in glycerol esterification with OA in the presence of different catalysts, namely, ZrO_2_-SiO_2_-Me&EtPhSO_3_H, Amberlyst 15 and Aquivion. Aquivion afforded the highest conversion, which was nearly 99% in 240 min reaction time (Figure 12A). Approximately 98% conversion was obtained within 120 min reaction time. The formation rates of GDO and GTO were the fastest for Aquivion (S_GDO_ = 69% and S_GTO_ = 30%), although an equimolar ratio of reactants was used. This result was attributed to the strong acidity of Aquivion. A relatively low selectivity of GMO was obtained for Aquivion-catalyzed reaction (< 3%). This finding suggested that the superstrong acidity of Aquivion is suitable in producing large GTO molecules at the OA-to-glycerol molar ratio of 3:1. Further lowering the loading amount of Aquivion (optimisation of catalyst concentration) is necessary to attain high yield and selectivity of GMO. Superstrong acid potentially produces undesirable side reaction products, ~50% by-product was attained in this experiment; the possible by-products would be glycerol oligomers, polyglycerol, alkenes, acrolein, or polyglycerol esters. A long reaction time is unadvisable for Aquivion-catalyzed reaction because the yield and GTO selectivity were reduced (Figure 12B).

Results showed that Amberlyst 15 obtained a higher yield and selectivity for GDO and GTO (S_GDO_ = 65% and S_GTO_ = 12%) than those of the two other catalysts; this result can be attributed to the lower acidity strength of Amberlyst 15 (H_0_ = −2) than that of Aquivion (H_0_ = −12) and its larger pore size (28.8 nm) than that of ZrO_2_-SiO_2_-Me&EtPhSO_3_H (3.77 nm). The undesirable by-product catalyzed by Amberlyst 15 is lesser (20%) than that of attained by Aquivion under identical reaction parameters. Prolonging the reaction time of Amberlyst 15–8 h increased the GDO and GTO selectivities (S_GDO_ = 74% and S_GTO_ = 16%). The water as a by-product from esterification reaction was reported can deactivate sulphonation exchanger of Amberlyst 15 by adsorbing water in its pores. It was reported addition higher amount of water in reaction resulted in slightly lower yield from 95 to 85% in synthesis of glucose esters using Amberlyst 15 (Ignatyev Igor et al., 2012).

Whereas, ZrO_2_-SiO_2_-Me&EtPhSO_3_H obtained the highest yield and GMO selectivity (60%), which proved that catalyst acidity is vital in controlling the conversion rate and yield. Firstly, a moderate acidity level of catalyst or suitable loading amount of catalyst is required to produce a high-yield product, and excess acidity may lead to side reaction. Secondly, textural properties, such as pore size/pore volume, influence the selectivity of a product significantly by controlling the pore size of catalyst to form the desired selectivity product. Catalyst recyclability and stability experiment of ZrO_2_-SiO_2_-Me&EtPhSO_3_H revealed that the yield decreased with the number of uses. The yield was reduced from 83, 74, and 69% in accordance with the number of times of usage. Herein, yield refers to the total GMO, GDO, and GTO in product mixtures, respectively. This trend may be attributed to that the GTO product blocks the active centers of the catalyst or the hydrophobic properties are lost (Zhang et al., 2017). The chromatogram peaks for products are provided in supplementary material (Figures S2, S3).

## Conclusions

In this work the esterification of oleic acid with glycerol was conducted on ZrO_2_-SiO_2_-Me&Et-PhSO_3_H catalyst. The reaction achieved 80% conversion with GMO and GDO being the major products having a combined selectivity of 94.8% (GMO = 59.4% and GDO = 34.6 %) at equimolar ratio of OA-to-glycerol, 160°C reaction temperature, 5 wt% catalyst concentration with respect to OA and for a reaction time of 4 h. After prolonging the reaction time to 8 h under the same operating parameters, 88.2% conversion with 53.5% GMO selectivity and 40.0% GDO selectivity (combined GMO and GDO selectivity = 94%) were obtained. It is found that increasing the reaction temperature accelerates the conversion rate but decreases the selectivity of GMO. The equimolar ratio of OA to glycerol was suggested to increase the selectivities of GMO and GDO. This work also confirmed that 5 wt% ZrO_2_-SiO_2_-Me&Et-PhSO_3_H catalyst concentration is the optimal level for the catalytic study of glycerol with OA. Moreover, the GMO selectivity decreased with the increased catalyst concentration. This effect was highly attributed to the increased number of available acidic sites of the catalyst.

Therefore, a strongly acidic catalyst promoted the formation of a low-GMO-selectivity product mixture. Comparison of the performance of ZrO_2_-SiO_2_-Me&Et-PhSO_3_H and commercially available Amberlyst 15 and Aquivion showed that catalyst acidity is a key parameter for catalytic activity and conversion rate. Nevertheless, high acidity/acid strength reduced the product yield in the glycerol esterification of OA. The mild acidity of ZrO_2_-SiO_2_-Me&Et-PhSO_3_H with a hydrophobic surface was recommended for the catalytic esterification of glycerol with OA at equimolar ratio of reactants to attain a high selectivity of GMO. Superacid Aquivion was recommended to produce GTO at an OA-to-glycerol moral ratio of 3:1. This study proved that the textural properties (pore volume and pore size); acidity and hydrophobicity of heterogeneous acid catalysts play vital roles in controlling the activity and selectivity of reactions. Therefore, the acid strength and the number of available acid sites influence the conversion rate of reaction, and the hydrophobicity and pore volume of solid catalysts significantly affect the selectivity of the product.

## Author Contributions

PSK and YP contributed to the conception and design of the study, organized the database and performed the statistical analysis. PSK wrote the first draft of the manuscript. YP, PC, WMAWD, and MKA contributed to the manuscript revision, read and approved the submitted version.

### Conflict of Interest Statement

PSK was employed by the company Sime Darby Research Sdn. Bhd., Malaysia. The remaining authors declare that the research was conducted in the absence of any commercial or financial relationships that could be construed as a potential conflict of interest.
