# Supplementary material for: Esterification of Glycerol With Oleic Acid Over Hydrophobic Zirconia-Silica Acid Catalyst and Commercial Acid Catalyst: Optimization and Influence of Catalyst Acidity
Source: Front Chem. 2019 Apr 10;7:205. doi: 10.3389/fchem.2019.00205 (PMC6477701; doi:10.3389/fchem.2019.00205)
Supplement: Supplementary file 1 [file Data_Sheet_1.docx]

**Supplemental material**

| 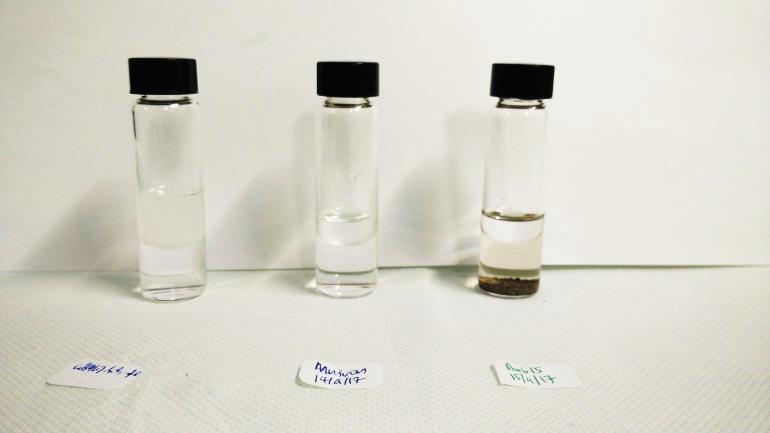  (a) | 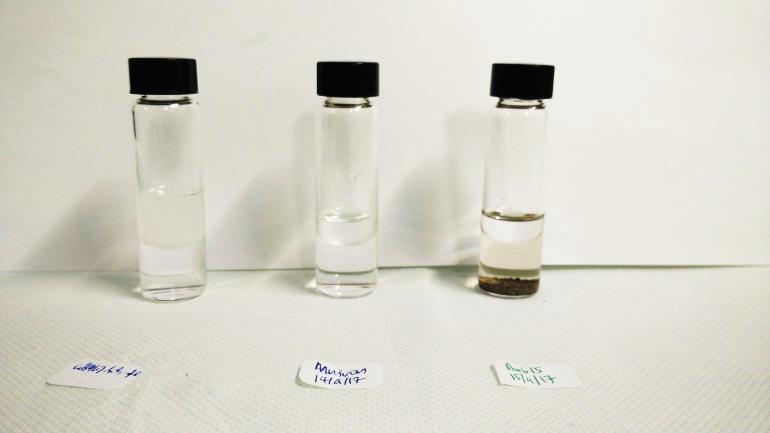  (b) | 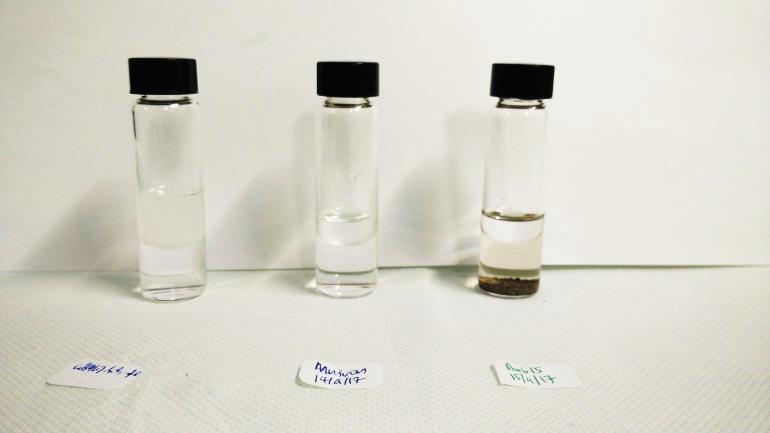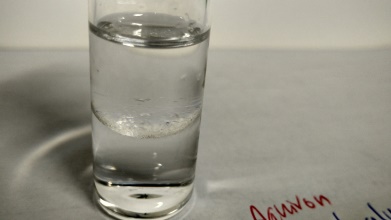  (c) |
| --- | --- | --- |

Figure S1: Sample photos of catalyst dispersed in toluene (top) and water (bottom): (a) Me&Et-PhSO_3_H-SiO_2_-ZrO_2_, (b) Amberlyst 15 and (c) Aquivion

|  |
| --- |

**Figure S2:** Chromatogram peaks for group of GMO and OA in 80ACN20H20 0.1%TFA mobile phase catalysed by ZrO_2_-SiO_2_-Me&Et-PhSO_3_H

|  |
| --- |

**Figure S3:** Chromatogram peaks for group of GDO and GTO in 40ACN40MeOH20THF mobile phase catalysed by ZrO_2_-SiO_2_-Me&Et-PhSO_3_H

Table S1: Comparison of the textural properties of ZrO_2_-SiO_2_-Me&EtPhSO_3_H catalyst with those of commercial Amberlyst 15 and Aquivion catalyst

| **Catalysts** | **BET** | | | **Acidity** | **Particle size distribution (µm)** |
| --- | --- | --- | --- | --- | --- |
|  | **Area (m^2^/g)** | **Pore volume (cm^3^/g)** | **Average pore diameter (nm)** |  |  |
| ZrO_2_-SiO_2_-Me&Et-PhSO_3_H | 79.75 | 0.0247 | 3.77 | 0.62 mmol/g | 5.01 |
| Amberlyst 15 | 42.5 | 0.290 | 28.8 | 4.7 mequiv./g | 300 |
| Aquivion PFSA- superacid* | <0.1 | - | - | 0.98- 1.06 mmol/g | 660 |

*The characterization data of Aquivion was obtained from [30]
